# Operation parameters investigation on combustion and emission of a non-road diesel engine based on orthogonal experiment design at different altitudes

**DOI:** 10.1016/j.heliyon.2024.e40086

**Published:** 2024-11-05

**Authors:** Zhaojun Song, Lianjiang Xu, Lan Zhang, Lun Zhao, Jin Ba, Wu Wei, Zeshan Abbas

**Affiliations:** a School of Mechanical and Electrical Engineering, Yunnan Open University, Kunming, 650000, China; b Shenzhen Polytechnic University, Shenzhen, 518055, China; c School of Mechanical Engineering, Guizhou University, Guiyang 550025, China

**Keywords:** Orthogonal experiment design, High attitude, Combustion and emissions, Operation parameters optimization

## Abstract

For improving the combustion and emission performance of engines operating at high altitude regions, a two OED optimization method was proposed. The influence of each factor on the target was analyzed and discussed through a primary OED. Based on the primary OED test, factors with less influence were excluded to reduce factors in the secondary OED test. The results showed that low intake pressure led to poor spray mixing, prolonged ignition delay, and incomplete combustion, resulting in deteriorated power output, fuel economy, and emission characteristics. After two OED optimizations, The NOx emissions increased significantly by 40.5 % and 122.5 % in the first and second optimization, respectively, however, the ISFC decreased by 13.4 % and 25.2 %, and CO decreased by 13.0 % and 25.2 % in the first and second optimization, respectively, as well as ultra-low carbon smoke emissions was achieved. The related theories and methods' advantage can be extended to engineering applications and has good practical value.

## 1 Introduction

Internal combustion engine is the main source power in transportation, agricultural, and industrial usage and plays a key role in the progress of human civilization and social development. Diesel engines are recognized as widely used in vehicles and non-road power machinery because of its high efficiency and power rise rate [[1], [2], [3], [4], [5]]. Although internal combustion engines bring convenience to human beings, its exhaust emissions also pose great challenges to ecological environment. The pollutants emitted by internal combustion engines mainly include nitrogen oxides (*NOx*), carbon monoxide (*CO*), unburned hydrocarbons (*HC*), and *SOOT* emissions, which pollute the atmospheric environment and have adverse effects on humans and ecosystems. Although *CO*_*2*_ is non-toxic, it is the main source of greenhouse gas. So, it has received extensive attention in recent years.

As the diesel engine works under high altitudes condition, the ambient pressure and temperature decrease, resulting in a reduction in air intake and oxygen concentration. Hence the pressure and temperature in-cylinder at TDC are lower, leading to the deterioration of fuel combustion, serious retarded combustion, and the pollutant emissions and exhaust temperature increase, while fuel economy decrease [6,7]. Relevant studies have shown that the power performance and fuel economy is decreased by 4.0 %–13.0 % and 2.7 %–12.9 % for every 1000m increase in altitude, respectively. Wherein, SOOT, *HC* and *CO* emissions increase by34 %, 30 % and 35 % respectively [8]. Other scholars have come to similar conclusions [[9], [10], [11], [12], [13], [14], [15], [16]]. According to the characteristics of diesel engine operating in high-attitude regions, the combustion performance inside cylinder obtains effective improvement by optimizing the combustion chamber structure and operation parameters, thereby improving the efficiency of fuel utilization and reducing exhaust emissions.

In terms of plateau internal combustion engine research, many scholars focused on the in-cylinder combustion process, emission control, fuel substitution and so on. In the early days, R.A.C Fosberry used the “all-climate laboratory” to establish a high-attitude condition and conducted experimental research on a variety of naturally aspirated diesel engines, aiming to calibrate the power of plateau diesel engines. JR Sodré and SMC Soares [17,18] systematically measured the operating conditions of the engine under different road, pressure, temperature and altitude conditions. The study tried to deduce the relationship between engine power and altitude. They pointed out that the drop in atmospheric pressure has the most significant impact on the performance of engine in high-altitude environments. The drop in pressure led to a reduction in air intake, resulting in the deteriorated combustion and a drop in power output, while the effects of temperature and humidity were almost negligible. In 2009, Agudelo et al. [19] analyzed the combustion and exergy characteristics of diesel engines at different altitudes, and found that the combustion temperature, heat transfer loss and exergy loss increased with altitude, while the thermal efficiency and in-cylinder pressure decreased. Granbosk and McCormick [20,21] conducted emission experiments on diesel at high altitudes, and the results showed that *PM*, *HC* and *CO* emissions showed an increasing trend, while the *NOx* emissions did not change significantly. For pollutant emission control, there have been many related studies in recent years. Piqueras et al. [22] established an engine including combustion, turbocharger and exhaust post-treatment models at high-altitude regions based on gas dynamics, and adopted boundary conditions to the exhaust post-treatment system to analyze the influence of filtration efficiency on catalyst conversion efficiency and particle filter performance. Ángel Ramos et al. [23] studied the influence of altitude on the actual driving performance, emissions and thermodynamic diagnosis of vehicles, and results showed that the altitude had no obvious impact on *HC* and *CO* emissions, while *NOx* emissions were about 10 times higher than the limit specified in European standards. Bermúdez et al. [24] investigated the synergistic effect of fuel consumption and post-treatment thermal management under low intake pressure conditions, and they believed that the correct calibration of pressurization pressure and EGR could reduce specific fuel consumption and increase the gas temperature of the exhaust post-treatment system. Under high altitudes environment, the oxygen concentration entering the engine cylinder decreases. Therefore, many scholars have turned to the research of oxygenated fuel to improve engine performance. Oxygenated fuels such as alcohols, ethers, and lipids are usually added to diesel fuel or gasoline, or used in neat form to improve combustion efficiency. Benjumea et al. [25] used pure palm bio-oil to investigate the combustion process of a HSDI diesel engine. Results showed that the combustion duration shortened while the pressure inside cylinder increased, and the reduction in thermal efficiency was relatively small. At the same time, the combustion speed was faster, and better engine performance could be obtained by burning pure palm bio-oil at high altitudes. Shen et al. [26] made different oxygenated fuel through mixing with different ratio of ethanol and biodiesel into diesel, and the effects of oxygen radio on engine dynamics, fuel consumption rate and soot emission were studied. The results showed that with increasing oxygen content, not significant difference was found in engine torque and fuel consumption rate, but the soot emission can be reduced significantly. Yan et al. [27], Liu et al. [28], Pedro et al. [15,[29], [30], [31], [32]] also studied the performance of high-altitude engine by fuel oxygen enrichment methodology. Boehman et al. [12] experimentally investigated the impact of Intake oxygen enrichment on diesel engine performance under high-altitude environments, and they suggested that Intake oxygen enrichment had a significant impact on peak temperature and engine power recovery.

At elevated altitude, the atmospheric pressure decreases by reduction of charge coefficient and deterioration of combustion, which in turn leads to the drop of power and the increase of emissions. For this reason, extensive studies were conducted to increase the charge coefficient. Yang et al. [33] studied the adaptability of several single-stage turbocharging systems at high-altitude regions, and developed a method that could be used for turbocharger and engine matching at different altitudes. The results indicated that variable geometry turbocharging (VGT) had good plateau adaptability for larger altitude conditions in terms of power recovery and BSFC. Li et al. [34] experimental matched the supercharged diesel engines operating under different altitudes environment. They designed a control method for the waste gate valve, and proposed a fuel compensation method to achieve power recovery. Deng et al. [35], Zhang et al. [36,37] and Hatami et al. [38] also carried out related research. In order to alleviate the decrease of air intake with increasing altitude, many scholars began to develop bipolar turbocharger to further improve the operation adaptability of diesel engine at high altitude. Zhang et al. [39,40] performed thermal balance experiments on adjustable two-stage turbocharged engines at different altitudes, trying to obtain the optimal adjustment strategy at different altitudes. Dong et al. [41] investigated the effect of VGT blade operating on low-speed matching of a two stage turbocharged diesel engine at different altitudes through simulation. Yang et al. [42] investigated the influence of altitude on the performance of the two stage turbocharging system of internal combustion engine through analysis and experimental methods, and proposed a new direction for the optimization and regulation method of the two stage turbocharging system. For more relevant studies for this research work, we have referred to the literature [33,35,[42], [43], [44], [45], [46], [47]].

Under low intake pressure conditions, engine performance can be improved by optimizing the air intake and fuel injection system [48]. The research content of this article is divided into two aspects, firstly, the influence of power, fuel economy, combustion and emission characteristics were investigated under different altitude conditions. Secondly, the initial temperature, injection advance timing, injection duration and fuel temperature were chosen as the optimization parameters at a specific altitude was optimized by two-stage OED orthogonal test method, which provided a theoretical basis for improving the comprehensive performance and emission characteristics of diesel engine at high-attitude region.

## 2 Governing equations and simulation models

The CONVERGE software has a significant advantage in the 3D unsteady calculation of spray, combustion, and flow in internal combustion engines. It can automatically generate high-quality orthogonal hexahedral meshes during simulation based on geometric model, saving a significant amount of time in mesh generation. The main governing equations are briefly introduced below in Eq. (1).

Continuity equation:

(1) $$\frac{\partial \rho_{m}}{\partial t}+\nabla \cdot \left(\rho_{m}u\right)=\nabla \cdot \left[\rho D\nabla \left(\frac{\rho_{m}}{\rho }\right)\right]+{\dot{\rho }}_{m}^{c}+{\dot{\rho }}^{s}$$

where $\rho_{m}$ is the density of species m, ρ is total density,*u* is velocity,*D* is diffusion coefficient, ${\dot{\rho }}_{m}^{c}$ and ${\dot{\rho }}^{s}$ are the chemically generated source term and spray source term, respectively. δ is the Dirac delta function, and ${\dot{\rho }}_{m}^{c}$ is calculated by Eqs. (2), (3):

(2) $$\frac{{\dot{\rho }}_{m}^{c}}{\rho }=\frac{dY_{k}}{dt}={\dot{\omega }}_{k}\nu W_{k}$$

Momentum equation:

(3) $$\frac{\partial \left(\rho u\right)}{\partial t}+\nabla \cdot \left(\rho uu\right)=\frac{1}{\alpha^{2}}\nabla p-A_{0}\nabla \left(2/3\rho k\right)+\nabla \cdot \sigma +F^{s}+\rho g$$

where *p* is the pressure, *α* is a dimensionless quantity, *A*_*0*_ is 1 in turbulent flow, σ is the viscous stress tensor, *F*^*S*^ is the rate of change of momentum per unit volume due to injection, and *g* is the specific volume force. *σ* can be defined as in Eq. (4):

(4) $$\sigma =\mu \left[\nabla u+{\left(\nabla u\right)}^{T}\right]+\lambda \nabla \cdot uI$$

*μ* and *λ* represent the viscosity coefficient and thermal conductivity, and*Ⅰ* represents the unit matrix as given in Eq. (5).

Energy equation:

(5) $$\frac{\partial \left(\rho I\right)}{\partial t}+\nabla \cdot \left(\rho uI\right)=-p\nabla \cdot u+\left(1-A_{0}\right)\sigma :\nabla u-\nabla \cdot J+A_{0}\rho \varepsilon +\overset{\cdot }{Q^{C}}+\overset{\cdot }{Q^{s}}$$

where*Ⅰ* is the specific internal energy, *J* is the sum of heat conduction and enthalpy diffusion, $\overset{\cdot }{Q^{C}}$ is the source term caused by the exothermic chemical reaction, $\overset{\cdot }{Q^{s}}$ is the source term produced by the spray. The heat flux *J* can be calculated from the following Eq. (6):

(6) $$J=-K\nabla T-\rho D\sum_{m}h_{m}\nabla \left(\rho_{m}/\rho \right)$$

where *T* and *h*_*m*_ represent fluid temperature and specific enthalpy of component *m*, respectively.

The RNG κ-ε turbulence model is applied in this simulation, and the ECFM-3Z flame model is adopted for combustion simulation. It divides the combustion process into air zone, mixed zone, and fuel zone, enabling the prediction of both premixed and diffused combustion. The “BLOB” model is applied to simulate the fuel block injection dynamics, while the K-H and R-T models proposed by Beale and Reitz [49] are used for droplet splitting and atomization. The rebound/slide model is used to simulate the droplet collision feature, including the bounce and slide of droplets after hitting the cylinder wall. The expanded Zeldovich model [50] is applied to predict *NOx* generation, taking into account the influence of free radical *OH* on *NOx* formation. Lastly, the two-step empirical Hiroyasu-NSC [51] model is utilized to predict soot emission, where the soot generation rate is calculated based on fuel concentration.

**Fig. 1:**
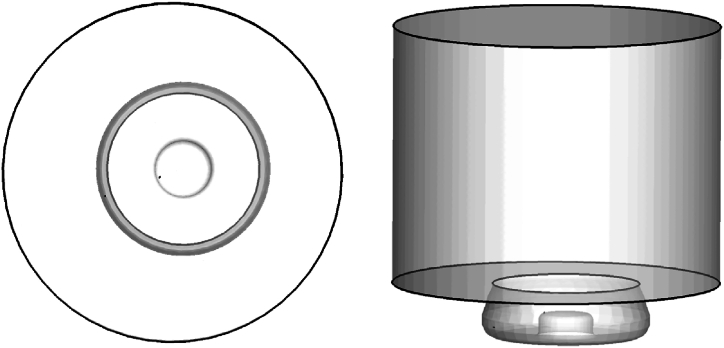
Cylinder model.

This paper focuses on studying a particular type of non-road naturally aspirated diesel engine running at different altitude regions. The engine's operating condition is presented in Table 1, with IVC and EVO times occurring at Before Top Dead Center (BTDC) 136 °CA and After Top Dead Center (ATDC) 132 °CA, respectively. Since the cylinder model is asymmetric, a complete cylinder model is required for the computational domain. Fig. 1 displays the cylinder geometry model, and the basic grid size is 4 mm.

**Table 1:** Operating parameters for engine.

**Parameters**	**Values**
Diameter	86 mm
Stroke	76 mm
Displacement	0.44(L)
Compression Ratio	19.0
Rated Power (kW)/(Speed)	6.8 (3600r/min)
Maximum Torque (N·m)	20.6 (2750r/min)
Number of Nozzle	5

At high altitude areas, the difference in atmospheric pressure and temperature significantly affects engine performance [52]. To study the in-cylinder combustion and emission characteristics of non-road diesel engines at different altitudes, altitudes of 10m, 1000m, 2000m, and 2500m are investigated. The atmospheric pressure at each altitude is calculated using formula (7), where p represents pressure (kpa), h represents altitude (m), and the temperature decreases by 7K for every 1000m increase in altitude. The initial pressure and temperature in the cylinder at different altitudes of 10, 1000, 2000, 2500 m are 100.1, 89.9, 79.5, 74.7 kpa and 320 313, 306, 303 K, respectively. To maintain the same power output at high altitudes, the amount of circulation fuel injection is increased. This study focuses on simulating and analyzing the combustion and emissions characteristics of diesel engines operating under different altitude regions as given in Eq. (7).

(7) $$p=e^{5.25885\times \ln\left(288.15-0.0065h\right)-18.2573}$$

## 3 Results and discussions

### 3.1 Model validation

**Fig. 2:**
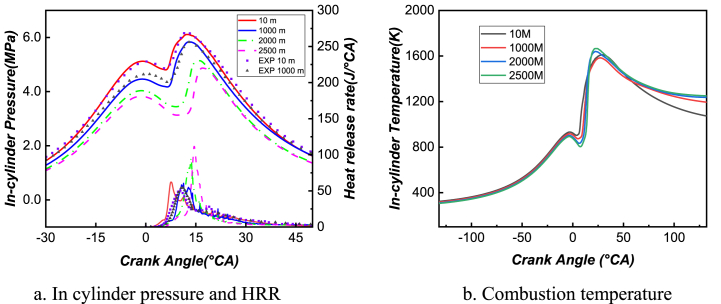
Variations of in-cylinder pressure, HRR, and combustion temperature versus crank angle at different altitude.

The combustion and emissions characteristics at different altitudes are simulated based on Table 2. Fig. 2 compares the simulated in-cylinder pressure, heat release rate, and temperature with experimental results. It is found that the simulated in-cylinder pressure and heat release rate curves are in good agreement with the measured results when the altitude is 10m and 1000m, although there is a certain degree of error between simulated values and experimental data that is mainly due to inevitable errors in experimental measurements and the simulation models, but the overall error is controlled within a reasonable range, guaranteeing the reliability of the model established in this study. The impact of altitude on cylinder pressure and temperature is studied by changing the initial and boundary conditions. As shown in Fig. 2(a), due to the small advancing injection angle, combustion occurs after TDC, resulting in a “double peak” cylinder pressure curve. The first peak value represents the pressure at the end of compression, and the second peak value is the maximum combustion pressure caused by the combustion exothermal stage. At high-altitude areas, the intake air decreases, while the fuel injection quantity increases, resulting in a richer mixture formation and deteriorated combustion, leading to a reduction of the maximum pressure inside the cylinder during the combustion process with increasing altitude. The peak phase of the heat release rate curve also shifts backward, indicating that the combustion timing continuously shifts backward with increasing altitudes, and the thermal efficiency of the engine decreases, so additional fuel is usually injected into the cylinder to compensate for the efficiency drop. Fig. 2(b) compares the combustion temperature inside the cylinder at different altitudes. Under low intake pressure conditions, the inflatable coefficient is small, resulting in lower compression pressure at TDC. To maintain constant power output, more fuel injection is required, leading to a larger combustion peak with increasing altitude (shown in the second peak). Furthermore, the exhaust temperature increases, resulting in significant energy loss. This is one of the reasons why the thermal efficiency of diesel engines decreases when operating at high altitude conditions.

**Table 2:** impact factors and level distribution.

**Number of levels**	**Influencing factor**			
	**A**	**B**	**C**	**D**
1	292	−6	12	330
2	302	−5	13	340
3	312	−4	14	350
4	322	−3	15	360

### 3.2 Combustion and emission characteristics

**Fig. 3:**
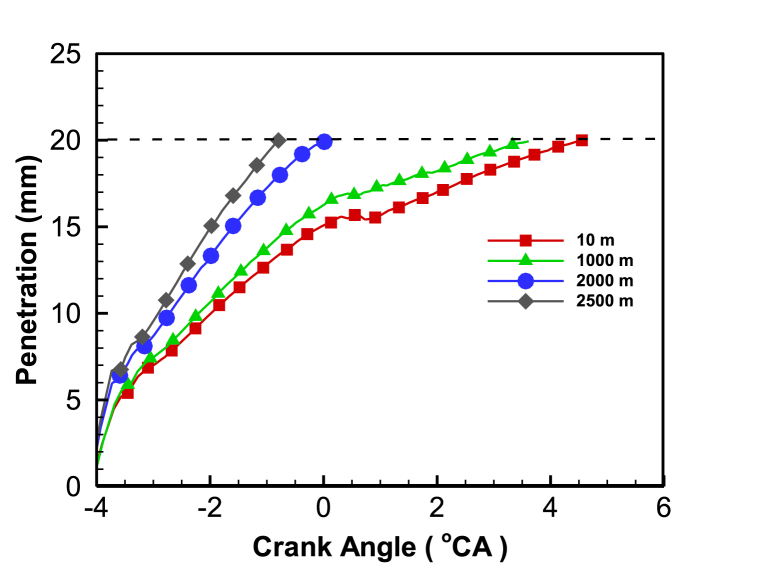
Penetration distance of diesel oil beam at different altitudes.

Fig. 3 displays the distribution of fuel spray penetration distance at different altitudes. The dashed line represents the distance to cylinder wall which is mainly determined by the combustion chamber's structural size. At high altitudes, the intake pressure decreases while the cylinder volume remains constant, reducing the density in the cylinder at the compression TDC, resulting in less resistance to the fuel bundle. As a result, the fuel beam tends to extend and expand in the direction of the spray, leading to longer spray penetration distances with increasing altitude. However, longer penetration distances make it easier for the oil bundle to hit the wall and result in shorter mixing times between droplets and air, which is not conducive to fuel evaporation. Consequently, fuel-rich mixtures tend to form in the spray liquid column and wall area. Additionally, due to the low gas density and temperature inside the cylinder, fuel bundle fragmentation slows down at high altitudes, leading to an increase in droplet diameter. This is also a significant reason for the poor fuel mixing in the cylinder under low intake pressure conditions.

**Fig. 4:**
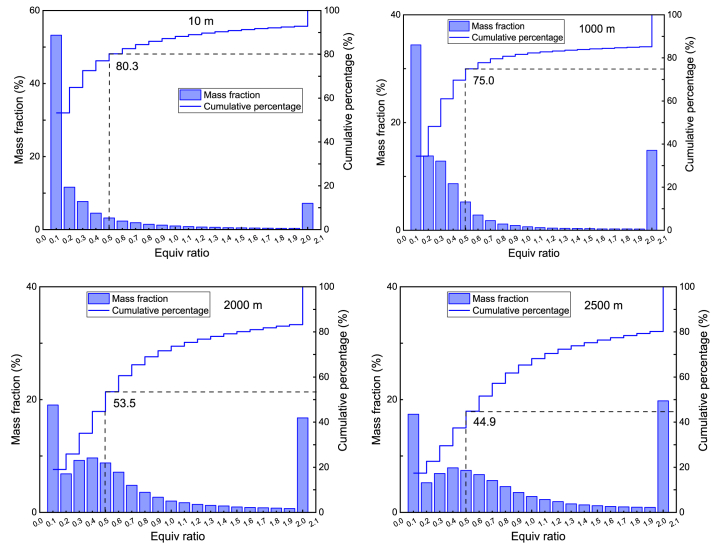
Mass percentage and cumulative mass of equivalence ratio ranges of the mixture in the cylinder at the moment of maximum heat release rate at different altitudes.

Fig. 4 shows the mass percentage and cumulative of mixtures with different equivalence ratios at the time of maximum heat release rate. Here, mixtures with equivalence ratios less than 0.5 are referred to as lean mixtures and marked in the figure. As shown in the figure, with the increase of altitude, on the one hand, the mass percentage of the lean mixture in the cylinder gradually decreases, and the equivalence ratio distribution gradually moves towards the rich mixture direction; on the other hand, the gradient of the cumulative percentage distribution of equivalence ratio in the cylinder increases, and the mixture formed in the cylinder becomes more inhomogeneous. At the moment of maximum heat release rate, the larger the mass ratio of lean mixture in the cylinder, the better the quality of fuel air mixture in the cylinder. At this time, the fuel combustion is more complete and the combustion efficiency of the fuel is higher. At high altitudes, the equivalence ratio in the cylinder increases, which is one of the necessary conditions for promoting the generation of soot.

**Fig. 5:**
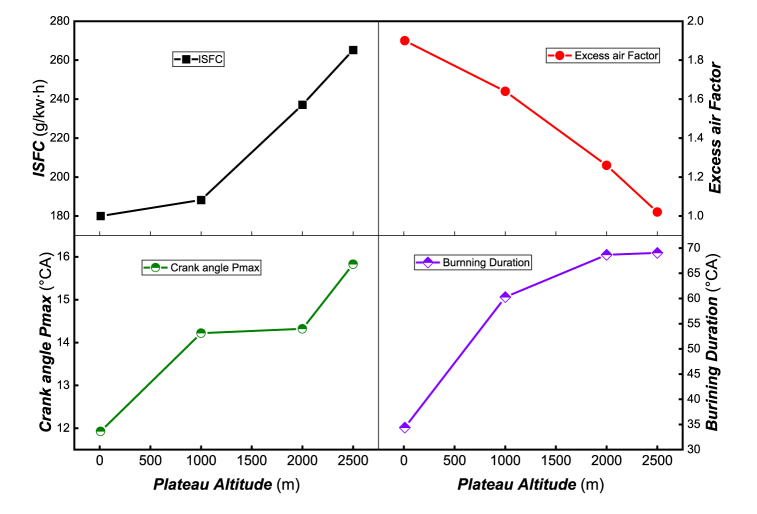
*ISFC*, excess air factor, crank angle of *P*_max_ and burning duration at different altitude.

Fig. 5 illustrates the indicative fuel consumption rate (*ISFC*), excess air coefficient, maximum pressure crankshaft angle, and combustion duration at different altitudes. At high altitudes, the atmospheric pressure drops, leading to a reduction in the air intake per cycle. To maintain a constant power output, the fuel injection must be increased while the excess air coefficient is decreased, resulting in poorer combustion and higher ISFC. As previously mentioned, in high altitude areas, the reduced air intake leads to a decrease in pressure and temperature, which delays the ignition timing. Consequently, the crankshaft angle corresponding to the highest combustion pressure is delayed with increasing altitude. To further investigate the combustion characteristics, the combustion initiation timing (*AI05*) and the combustion duration (*BD*) are defined. *AI05* is defined by *CAD* corresponding to 5 % of the cumulative heat release achieved. The combustion duration (*BD*) is defined by the *CAD* interval between 5 % and 90 % of the cumulative heat release. As shown in Fig. 5, the combustion duration (*BD*) widens with the increase of altitude, adversely affecting fuel economy and emission characteristics.

**Fig. 6:**
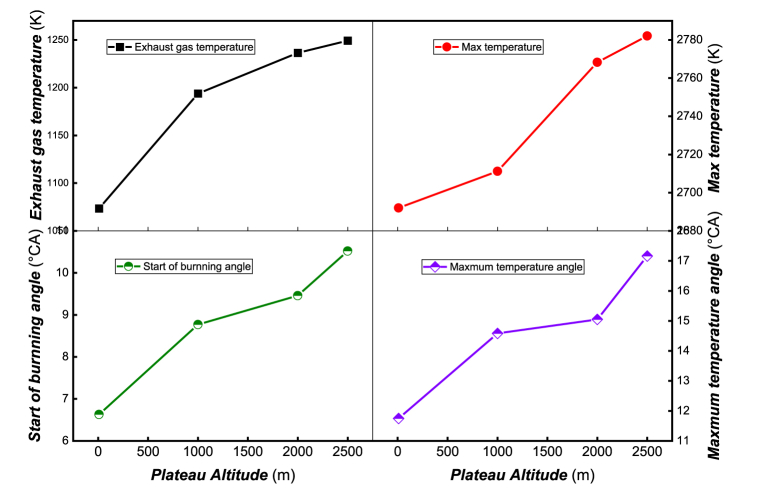
Exhaust temperature, maximum temperature, start burning angle and maximum temperature angle at different altitudes.

Fig. 6 illustrates the distribution of exhaust temperature, maximum temperature, start burning angle, and maximum temperature angle at different altitudes. As altitude increases, the exhaust temperature gradually rises due to the more significant afterburning proportion. Meanwhile, to maintain the same power output, the amount of fuel injection must increase, compensating for the decrease in pressure by raising the temperature. Consequently, the maximum combustion temperature in the cylinder increases with altitude. The ignition timing and combustion process are controlled by chemical dynamics, which are closely related to the pressure, temperature, and composition in the cylinder. As shown in the figure, with increasing altitude, the ignition timing is delayed, and the ignition delay period which is dominated by both physical and chemical properties is prolonged, resulting in the formation of a large amount of combustible mixture. This leads to the generation of a higher combustion temperature, and the location of the highest combustion temperature shifts backward.

**Fig. 7:**
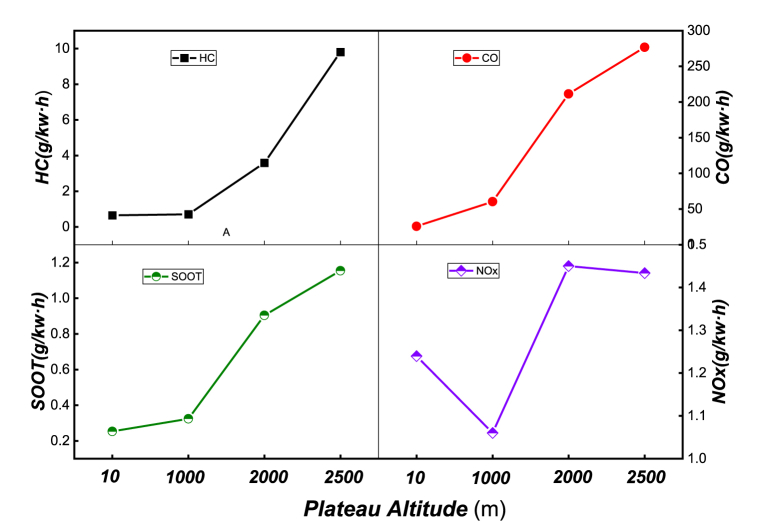
Emission of *CO*, *HC*, *NOx* and *SOOT* at different altitudes.

Fig. 7 displays the distribution of *CO*, *HC*, *NOx*, and *SOOT* emissions at different altitudes. The figure shows that *CO* emissions increase sharply, mainly due to the reduction of air intake with increasing altitude. This leads to the formation of a large number of fuel-rich areas, resulting in incomplete combustion. The increase in *HC* emissions is even more pronounced, mainly due to the following reasons: (1) A thin mixing area is formed outside the fuel spray column. Furthermore, as the air intake pressure decreases and the pressure and temperature at the end of compression decrease, incomplete combustion of the thin mixture far from the ignition area occurs, leading to a rapid increase in *HC* emissions. (2) At high altitudes, the insufficient oxygen in the cylinder hinders the further oxidation of *HC*, leading to an increase in *HC* emissions. (3) As altitude increases, the pressure in the cylinder decreases, causing the spray tip penetration to increase while the spray cone angle is reduced, resulting in poor fuel atomization quality. The lean mixture area increases the probability of incomplete combustion and flame quenching, leading to an increase in *HC* emissions.

As altitude increases, soot emissions gradually rise. From 10m to 1000m, there is little difference in soot emissions, but from 1000m to 2000m, soot emissions increase sharply. This is mainly because at high altitude, the amount of intake air decreases, resulting in a denser gas mixture and generating a large amount of soot during the combustion process. Furthermore, at high altitudes, the oxygen concentration decreases, constraining the oxidation effect on soot. Therefore, a sharp increase in soot occurs under low intake pressure conditions. Unlike the formation mechanisms of *CO*, *HC*, and *SOOT*, *NOx* is mainly controlled by combustion temperature, oxygen concentration, and high-temperature residence time. When altitude increases from 10m to 1000m, *NOx* emissions decrease slightly, while at 2000m, *NOx* emissions increase slightly, but the difference is not significant. The primary reason lies in the increase in altitude and the maximum average temperature in the cylinder, resulting in a stronger *NOx* production effect than the inhibition of *NOx* generation due to hypoxia, causing a slight increase in *NOx* generation. When altitude rises to 2500m, *NOx* emissions decrease slightly due to the inhibition of *NOx* generation resulting from the reduction of oxygen concentration.

### 3.3 Orthogonal experiment and optimization

Based on the analysis above, the increase in altitude leads to a decrease in atmospheric pressure, resulting in insufficient air intake, reduced combustion efficiency, and increased pollutant emissions. Orthogonal experimental design (*OED*) is commonly used to investigate multi-factor and multi-level problems. Some representative points are selected from comprehensive experiments based on orthogonality, which have uniform dispersion, neatness, and comparability. *OED* is a primary method of fractional factorial design and is widely used in various fields [[53], [54], [55], [56]]. By analyzing representative experiments, the degree of influence of factors on the target value can be determined, and parameter combinations close to the optimum can be obtained. For instance, if there are four variables in the test, with each variable having four levels, a complete test would require 4^4^ = 256 groups of tests to be conducted based on the permutation and combination. However, this is almost impossible in engineering applications with large M or F. Therefore, the basic idea of orthogonal testing is to choose a representative subset of all combinations, which greatly reduces the number of tests. In the *OED* experiment, orthogonal tables are initially designed based on different F and M. Generally, orthogonal arrays are defined as in Eq. (8):

(8) $$L_{R}\left(m^{F}\right)={\left(a_{i,j}\right)}_{R\times F}$$

Here $L_{R}\left(m^{F}\right)$ represents matrix, while *m*^*F*^,*R* denote the level combinations in the complete factorial experiment and number of trials, respectively.

Range analysis has the advantages of convenient calculation, intuitiveness, and ease of understanding, and is the most commonly used method in orthogonal experimental analysis. *R*_*j*_ represents the range of the factors in column *j*, which is the maximum and minimum difference of the index values at each level of the factors in column *j* as given in Eq. (9):

(9) $$R_{j}=\max\left({\bar{K}}_{j1}{\bar{K}}_{j2},...,{\bar{K}}_{jm}\right)-\min\left({\bar{K}}_{j1}{\bar{K}}_{j2},...,{\bar{K}}_{jm}\right)$$

Where *K*_*jm*_ represents the total of test values corresponding to the factor M for column *j*, ${\bar{K}}_{jm}$ represent the average value, which is used to judge the optimal level of factor *J* and the optimal combination of all factors, namely optimal parameter combination. *R*_*j*_ reflects the level change of the factor in column *j*. The larger the change range of *R*_*j*_ indicates the greater the influence of this factor on the test. According to the size of the range *R*_*j*_, the arrangement relation of the factors is obtained.

In this study, an *OED* is conducted on an engine with a speed of 3600r/min at 2500m. The intake air temperature (A), injection timing (B), injection duration (C), and fuel temperature (D) are crucial factors that affect the comprehensive performance and emission characteristics. Therefore, they are chosen as factors for the orthogonal experiment, where each factor can be divided into four levels based on the original diesel engine parameters as shown in Table 2. After determining the parameters, the orthogonal test table generated using SPSS software and results of orthogonal experiment are presented in Table 3.

**Table 3:** Orthogonal test scheme and results of orthogonal experiment.

**Exp**	**A**	**B**	**C**	**D**	***ISFC***	***CO***	***NOx***	***SOOT***
1	292	−6	12	330	265.25	240.9	2.02	0.455
2	292	−5	13	340	286.79	284.9	1.48	0.656
3	292	−4	14	350	307.46	296.7	0.95	0.763
4	292	−3	15	360	341.73	315.8	0.60	0.596
5	302	−6	13	350	279.95	285.0	2.50	1.051
6	302	−5	12	360	272.75	268.8	2.51	0.700
7	302	−4	15	330	314.27	314.8	1.38	1.510
8	302	−3	14	340	321.36	333.7	1.06	1.161
9	312	−6	14	360	288.63	316.1	2.96	1.304
10	312	−5	15	350	298.67	340.5	2.58	1.532
11	312	−4	12	340	286.60	298.7	2.44	0.915
12	312	−3	13	330	311.36	344.8	1.86	1.233
13	322	−6	15	340	299.99	338.3	2.93	1.551
14	322	−5	14	330	304.36	354.4	2.92	1.524
15	322	−4	13	360	296.60	326.1	3.12	1.117
16	322	−3	12	350	292.70	306.3	2.56	0.887

**Fig. 8:**
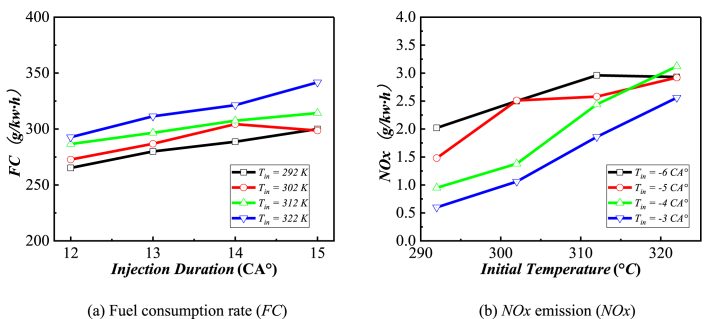
Evolutionary trends of C and A on the Fuel consumption rate and *NOx* emission.

The range analysis in Table 4 reveals that the injection duration (C) and intake temperature (A) are the key factors impacting *FC.* Therefore, the influence of injection duration (C) and intake temperature (A) on *FC* are displayed in Fig. 8(a). From the overall distribution, increasing the inlet temperature and injection duration are not conducive to reducing fuel consumption, so the smallest inlet temperature and injection duration corresponding to the lowest fuel consumption. Interestingly, at inlet temperature equal to 302*K* and the injection duration is increased from 14 to 15 °CA, the fuel consumption decreased instead. By observing the range analysis in Table 5, the injection timing likewise contributes to the fuel consumption. The mainly impact of injection timing (B) and inlet temperature (A) on *NOx* are shown in Fig. 8(b). It is discovered that the *NOx* emission increased with inlet temperature accepted for injection timing = −5 °CA, which can be contributes to injection duration (C). Similarly, advancing the injection timing (B) has adverse effects on *NOx* emissions.

**Table 4:** Range analysis.

	Index	A	B	C	D
R_j_	CO	46.700	30.075	48.675	7.200
	FC	3.993	33.333	34.340	5.233
	NOx	1.620	1.083	0.509	0.320
	SOOT	0.652	0.134	0.558	0.252

Note: the bolded font is the maximum value in a line.

**Table 5:** Impact factors and level distribution.

**Number of levels**	**Influencing factor**		
	**A**	**B**	**C**
1	290	−8	10
2	295	−7	11
3	300	−6	12

**Fig. 9:**
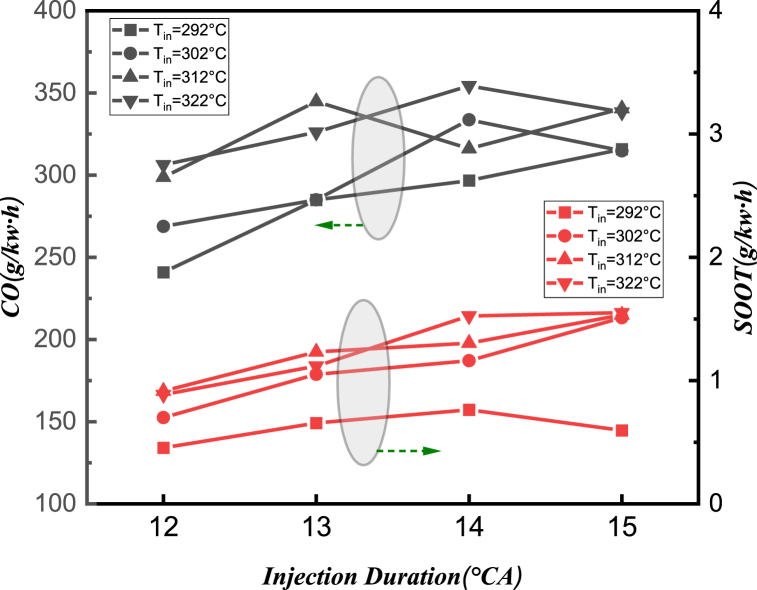
Evolutionary trends of A and C on the *CO* and *SOOT* emission.

Similarly, the range analysis reveals that the inlet temperature (A) and injection duration (C) have an important influence on *CO* and *SOOT* emission. As shown in Fig. 9, when the injection duration stays at 12 °CA, both the *CO* and *SOOT* emission increase with the increase of intake temperature. At the same time, by observing the *SOOT* emission, the overall trend is that the emission of *SOOT* almost increases with the increase of intake temperature, indicating that the *SOOT* emission is closely related to oxygen concentration. However, *CO* emission is generally not only related to intake temperature, but also to fuel injection pulse width. Take the inlet temperature (A) = 312 °C for example, *CO* emissions reach their maximum at a fuel injection pulse width of 13 °C, then significantly decrease at 14 °C, while increase again at 15 °C. This is because the injection timing also has an impact on *CO* emission, as demonstrated in Table 5.

**Fig. 10:**
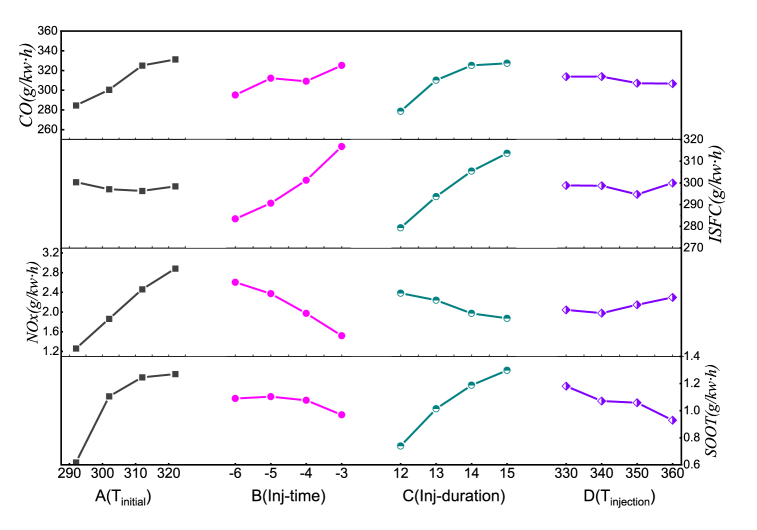
The effect curves of different level pairs of factors on each index.

Effect curves are used to reveal how the independent factors affect the target values. Fig. 10 displays the influence of each factor on the index at different levels. The *CO* and *SOOT* are sensitive to inlet temperature (A) and injection duration (C) with a larger curve slope. This can be attributed to lower inlet density at higher inlet temperature and the fuel injected into the cylinder in the later stage do not fully burn due to lack of oxygen at large injection duration. However, the influence of fuel injection advance angle and fuel temperature is relatively limited. The *ISFC* curve has a larger slope at different injection timing (B) and injection duration (C), indicating that these two factors pose a key impact on *ISFC.* While the inlet temperature and injection timing play an important role on *NOx* emission, which is owing to the high combustion temperature in the cylinder. Increasing the intake temperature and advance injection timing will lead to an increase in combustion temperature, which will be beneficial for the generation of *NOx*. Interestingly, emissions are sensitive to intake temperature, while fuel temperature has little effect on all indicators.

**Fig. 11:**
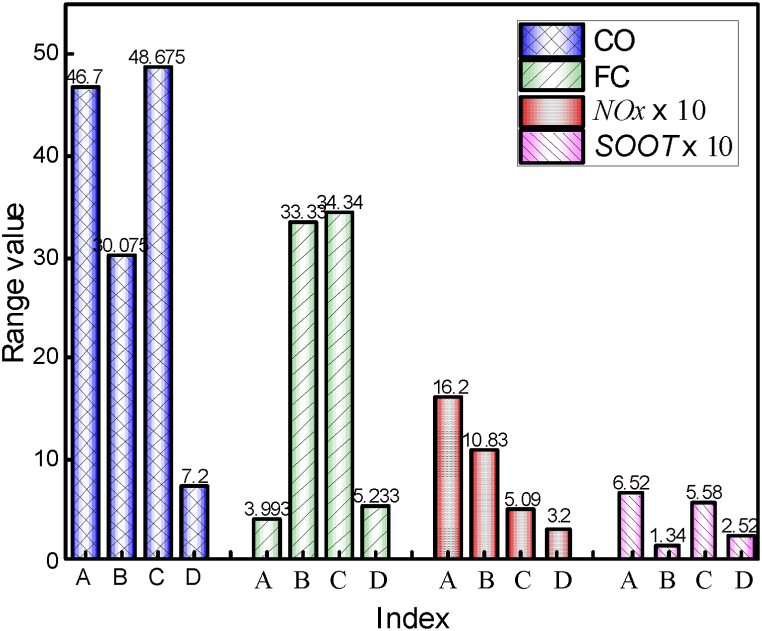
The importance of influencing factors on *CO*, *ISFC*, *NOx* and *SOOT*.

In order to analyze the factor, influence on targets more accurately, a range analysis is carried out for the orthogonal experiment results. The range is the difference between the maximum and values of all levels of a certain factor, and the range value represents the influence of the factor on the target value. Fig. 11 shows the range analysis results of each factor on *CO*, *FC*, *NOx* and *SOOT*. Range analysis shows that the injection duration (C) and the initial temperature (A) significantly impact *CO* emissions, while the fuel temperature (D) has the least effect. The order of influence of each factor on *CO* emissions is C > A > B > D. For *FC*, the fuel injection timing (B) and injection duration (C) have a more significant impact, while the fuel temperature (D) has the least. Thus, the effect of fuel temperature (A) on *CO* and fuel consumption rate is secondary and can be disregarded in the optimization analysis. It is found that the initial temperature (A) has the most significant impact on *NOx* emissions, whereas the fuel temperature (D) has a lesser influence, and the order of influence of each factor on *NOx* emission is A > B > C > D. For *SOOT* emissions, the initial temperature (A) has the most significant influence, whereas the injection timing (B) has the least, and the order of influence is A > C > D > B. Thus, the effect of fuel temperature on *NOx* and *SOOT* is secondary and can be disregarded in the optimization process.

### 3.4 Quadratic orthogonal experiment optimization

To achieve a superior optimization scheme, a new target value is obtained by weighted averaging over all target values (see Table 3). A comprehensive range analysis is conducted on these new target values and found that the factors influencing the optimization scheme are in the order of A > C > B > D. The optimal scheme is identified as A_1_B_1_C_1_D_1_, meaning that the initial temperature should be set at 292 K, the injection timing angle at −6 °CA, the injection duration at 12 °CA, and the injection temperature at 340 K. it is evident that the fuel injection temperature has minimal impact After the previous orthogonal test optimization. So, it can be excluded from the second orthogonal test for saving computing resources. For the second orthogonal test, only the fuel initial temperature (A), injection timing (B) and injection duration (C) are considered as main factors. It has three levels as outlined in Table 5. The orthogonal test table and range analysis are presented in Table 6, Table 7 respectively.

**Table 6:** Orthogonal test table results of orthogonal experiment.

**Exp**	**A**	**B**	**C**	***ISFC***	***CO***	***NOx***	***SOOT***
1	290	−8	10	229.06	132.4	3.19	0.028
2	290	−7	11	242.46	171.8	2.40	0.136
3	290	−6	12	263.67	228.7	1.91	0.353
4	295	−8	11	240.26	170.2	3.19	0.136
5	295	−7	12	253.58	207.5	2.51	0.417
6	295	−6	10	238.10	148.3	2.53	0.093
7	300	−8	12	246.28	187.5	3.08	0.330
8	300	−7	10	238.53	158.3	3.15	0.110
9	300	−6	11	251.90	193.8	2.73	0.319

**Table 7:** Range analysis.

	Index	A	B	C
R_j_	CO	4.534	26.900	61.567
	FC	1.590	12.690	19.280
	NOx	0.487	0.763	0.457
	SOOT	0.081	0.090	0.290

Note:the bolded font is the maximum value in a line.

**Fig. 12:**
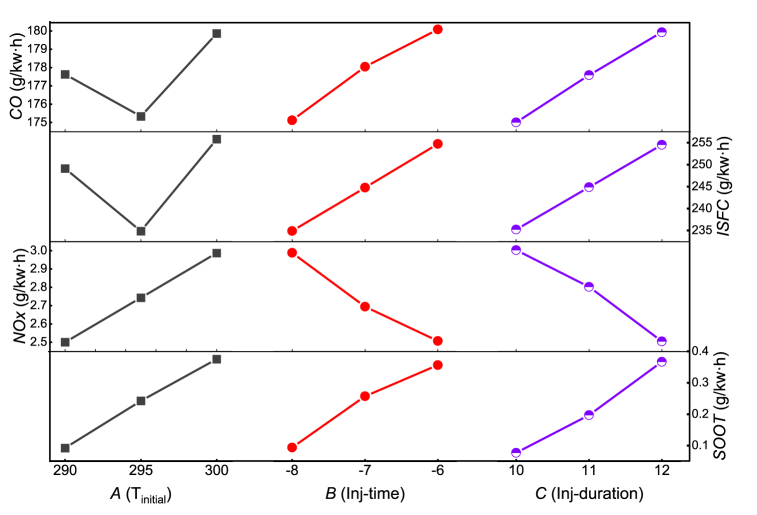
The effect curves of different level pairs of factors on each index.

Fig. 12 shows the influence of independent factors on the index at different levels. The *NOx* and *SOOT* emission monotonically increasing with the increase of intake temperature due to the lower intake density causing incomplete combustion and higher combustion temperature in cylinder. However, the impact of initial temperature on *CO* and *ISFC* is not consistent. For injection timing (B) and injection duration (C), the trend of their impact on all target values is almost the same, that is, delaying the injection advance angle and increasing the injection pulse width are unfavorable for *CO*, *ISFC* and *SOOT*, while on the contrary, they are favorable for *NOx* emissions. This also proves that *NOx* and *SOOT* have a trade-off relationship due to the production mechanisms are different. Under high temperature conditions, *CO* is easily oxidized and reduced, but *NOx* is more easily generated in high-temperature oxygen-rich environments. Obviously, advancing the injection timing and shortening the injection pulse width can reduce fuel consumption and other emissions, but it will lead to an increase in *NOx* emissions. In addition, through two orthogonal experiments, it is found that an increase in intake temperature has almost adverse effects on fuel consumption and emissions, which is why an intercooler is needed after turbocharging.

**Fig. 13:**
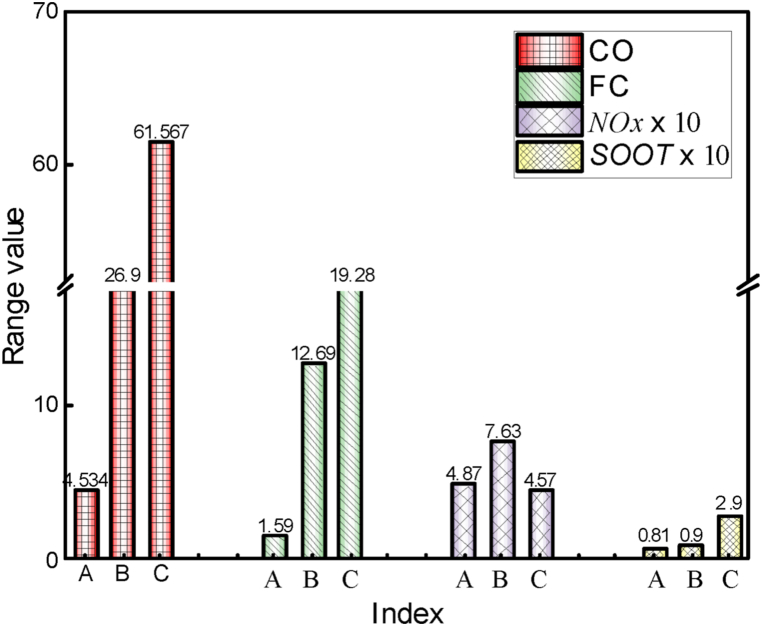
The importance of influencing factors on *CO*, *ISFC*, *NOx* and *SOOT*.

The range analysis in Fig. 13 reveals that the injection duration (C) has a significant influence on *CO* emissions, whereas the initial temperature (A) has a lesser impact. The order of influence of various factors on *CO* emissions is C > B > A. The effects of injection duration (C) and injection timing (B) on *ISFC* are greater than those of the initial temperature (A). From the primary and secondary orthogonal tests, the injection duration plays a crucial role in *ISFC* and *CO* emissions.

### 3.5 Selection and verification of optimization scheme

**Fig. 14:**
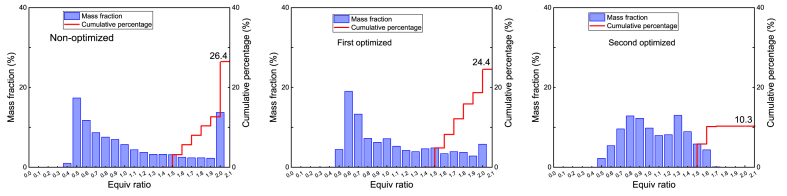
Mass percentage distribution of different equivalence ratio ranges of the mixture at 132 °CA under non/first/second optimization.

After conducting two times of *OED* and considering *SOOT* and *CO* emissions as optimization goals, the final optimized scheme is A_1_B_1_C_1_, with the initial temperature being 290K, the fuel injection timing being −8, and the injection duration being 10 °CA. Fig. 14 Mass percentage distribution of different equivalence ratio ranges of the mixture at 132 °CA under non/first/second optimization. A mixture with an equivalence ratio greater than 1.3 is called a rich mixture, and its cumulative mass percentage is indicated in the figure. After one and two optimizations, on the one hand, the proportion of dense mixed gas in the cylinder is reduced, and the distribution of equivalence ratio becomes more uniform; On the other hand, the cumulative mass percentage of the dense mixed gas decreases (non-optimized: 26.4 %, first optimized: 24.4 %, second optimized: 10.3 %). From the mechanism of pollutant generation, it can be seen that the fuel-air distribution in the cylinder has a significant impact on the formation of emitted pollutants. Through two OEDs, the proportion of rich mixture in the cylinder is reduced, which is an important condition for promoting the generation of soot.

**Fig. 15:**
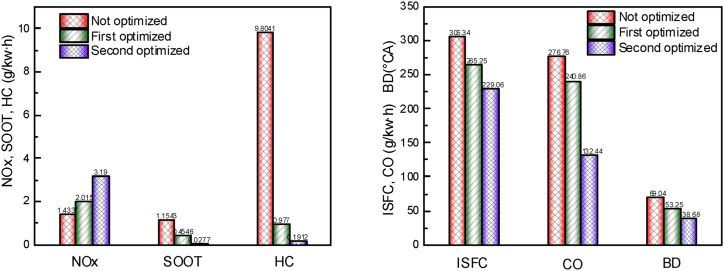
Comparison of emission characteristics, fuel efficiency and combustion duration after first and second optimization.

Fig. 15 shows the *NOx*, *SOOT*, *HC*, and *CO* emissions, as well as indicated fuel consumption and combustion duration at non-optimized, first optimized and second optimized stages. It is observed that a trade-off exists between *NOx* and *SOOT* as well as between *NOx* and *ISFC* after the optimization of the two *OED*s. To compensate for insufficient cylinder pressure, it is necessary to increase cylinder temperature and improve engine power characteristics and emission characteristics under high stall conditions due to insufficient intake air. So, the *NOx* emissions increased significantly in the first and second optimization, reaching 40.5 % and 122.5 %, respectively. However, the optimization of other parameters achieved good results, with significant reductions in *SOOT* and *HC* emissions after the first and second optimization. Additionally, *ISFC* and *CO* also achieved positive effects after optimization with *ISFC* decreasing by 13.4 % and 25.2 %, and *CO* decreasing by 13.0 % and 25.2 % in the first and second optimization, respectively. The combustion duration significantly affects fuel economy and emission characteristics, and it is found to be shortened during the optimization process, which is beneficial in reducing fuel consumption and pollutant emissions. This is mainly because the shortening of the combustion duration leads to an increase in the combustion heat release rate and maximum combustion temperature, which promotes the formation of *NOx*. However, since the generated *NOx* is not fully oxidized with the piston moving down, this ultimately leads to an increase in *NOx* emissions.

**Fig. 16:**
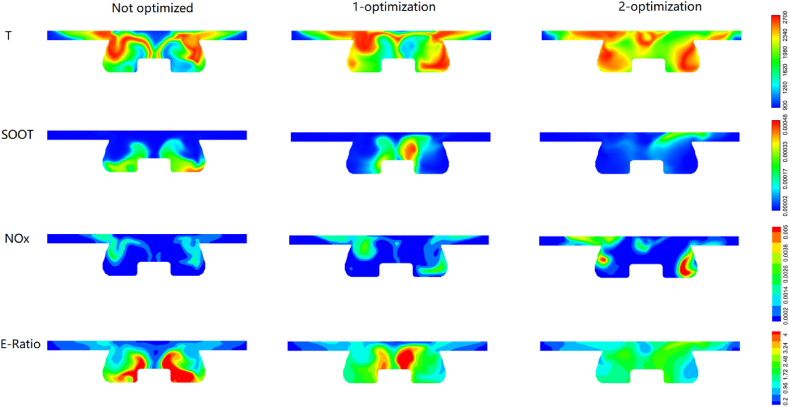
Distribution of in-cylinder temperature, *NOx*, *SOOT* and equivalence ratio at 20 CA at non-optimized, 1-optimization and 2-optimization (the red and blue color corresponding to high and low value).

**Fig. 17:**
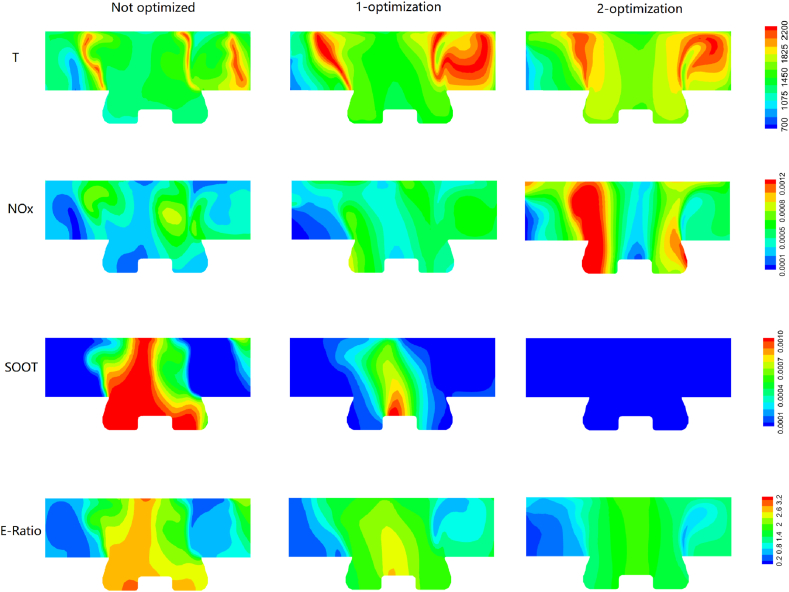
Distribution of in-cylinder temperature, *NOx*, *SOOT* and equivalence ratio at 60 CA at non-optimized, 1-optimization and 2-optimization (the red and blue color corresponding to high and low value).

Fig. 16, Fig. 17 illustrate the distributions of in-cylinder temperature, *NOx*, *SOOT* and equivalent ratio before and after *ODE* optimization at 20 °CA and 60 °CA, respectively. At 20 °CA, due to the increase in fuel injection timing and the decrease in injection duration, the ignition delay and temperature increase significantly. The un-optimized operating condition has a smaller high temperature area than the secondary *ODE*, and there are more high temperature areas in the secondary optimization than the un-optimized condition. The generation of *SOOT* is significantly affected by temperature and equivalent ratio. Due to the advance of fuel injection, the contact time between fuel and air increases, resulting in the formation of more uniform mixtures and fewer large equivalent ratio areas that inhibit the generation of *SOOT*. However, a trade-off exists between *NOx* and *SOOT*, and the emission of *NOx* increases in the secondary ODE. As the piston moves down to 60 °CA, both the pressure and temperature in the cylinder decrease, while the secondary ODE still contains a large high-temperature region, which is the main reason for *NOx* generation. Upon careful observation, it is found that a large equivalent ratio is formed at the bottom of the piston under non-optimized conditions, leading to a significant amount of soot generation by hypoxic combustion. However, the distribution of the equivalent ratio is relatively uniform and small after two ODE optimizations, resulting in less soot generation at this stage.

**Fig. 18:**
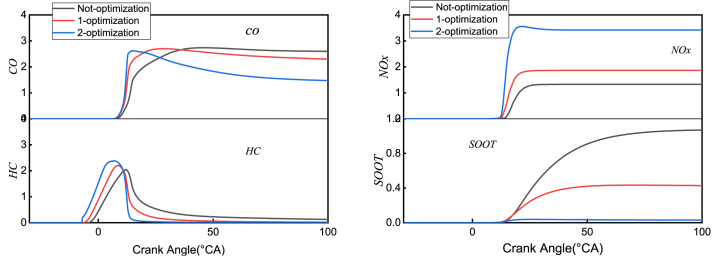
*CO*、*HC*、*NOx*、*SOOT* curves at non-optimization, 1-optimization and 2-optimization.

Fig. 18 displays the formation processes of *CO*, *HC*, *NOx*, and *SOOT* emission pollutants before and after optimization. Through the above analysis, it is observed that after the secondary optimization, the relatively low air intake temperature results in an increase in the amount of air entering the cylinder. Additionally, the fuel injection advance and smaller injection duration lead to earlier *CO* generation and a smaller peak value. Since the equivalence ratio distribution is relatively uniform and small, the generated *CO* is oxidized at a faster rate in the later stage, resulting in a rapid decrease in the *CO* curve. However, the peak value of *HC* reaches its maximum, mainly due to the reduction in injection duration and a large amount of fuel injected into the cylinder in a short period, resulting in low temperature and oxygen deficiency. As the combustion continues, the generated *HC* is oxidized and consumed, and its oxidation mechanism is consistent with that of *CO*, where the equivalent ratio plays an essential role in the formation of both *HC* and *CO*. The distribution of *NOx* gradually increases in the primary and secondary optimizations due to its easier generation under high-temperature and oxygen-enriched conditions. In the primary and secondary *ODE* optimizations, the fuel injection is advanced, the ignition delay period is prolonged, and the air intake temperature is low, resulting in an increase in air density, which creates favorable conditions for the generation of *NOx*. However, the *SOOT* distribution curve is just the opposite. After the optimization, the formation of *SOOT* is suppressed under the high-temperature and oxygen-enriched condition, leading to a significant decrease in *SOOT*. At the same time, the oxygen-enriched condition plays a positive role in the oxidation of *SOOT* in the later stage. Therefore, after the second optimization, *SOOT* decreased significantly, mainly because after secondary *OED* optimization, the proportion of large equivalence ratios is significantly reduced (Fig. 14). Upon careful observation, it is found that as the piston moves to a certain stage, the distribution of *NOx* and *SOOT* remains relatively unchanged, that is a “freezing” phenomenon occurs, indicating that the generation and oxidation of *NOx* and *SOOT* have reached an equilibrium state.

## 4 Conclusions

This paper comprehensively examines the combustion and emission characteristics of a non-road diesel engine at different altitudes of 10m, 1000m, 2000m, and 2500m using numerical methods. A two *OED* is also applied to analyze the primary and secondary relations among the factors, as well as an optimized scheme is obtained. The main conclusions are drawn as follow.

(1) With the increase of altitude, atmospheric pressure decreases, resulting in insufficient air intake, longer injection penetration distance, and poor atomization. This ultimately leads to worse combustion and delay combustion ignition timing, resulting in a serious after-combustion phenomenon. This not only decreases power output, but also increases pollutant emissions.
(2) The duration of injection has the most significant impact on *CO* emissions, whereas both the timing and duration of injection play a decisive role in *ISFC.* The emissions of *NOx* and *SOOT* are highly sensitive to the temperature of the air intake. In contrast, the fuel temperature has minimal effect on engine emissions and fuel economy.
(3) Through two-stage *OED* optimization, the combustion duration is shortened, and fuel economy is effectively improved. In terms of emission characteristics, except for *NOx*, significant reductions are observed in *CO*, *HC*, and *SOOT* emissions.
(4) After *OED* optimization, a relatively uniform distribution of equivalence ratios is obtained and the proportion of large equivalence ratios is significantly reduced. As a result, only a minimal amount of *SOOT* is produced in this work.

Overall, the optimized parameters can be obtained with fewer computer resources by using two-stage OED optimization and the impact of each parameter and optimal parameter combination can be obtained. This research method can be applied to many engineering research and application fields and has good practical value.

**Table:** Nomenclature

OED	Orthogonal Experiment Optimization	BD	burning duration
ISFC	Indicated Specific Fuel Consumption	SOC	starts of combustion
CA	Crank Angle	BTDC	Before Top Dead Center
IVC	Intake Valve Closing	ATDC	After Top Dead Center
EVO	Exhaust Valve Closing	ICP	In-Cylinder Pressure
CAD	Crankshaft angle		

## CRediT authorship contribution statement

**Zhaojun Song:** Writing – original draft. **Lianjiang Xu:** Writing – review & editing. **Lan Zhang:** Methodology. **Lun Zhao:** Investigation, Funding acquisition. **Jin Ba:** Visualization, Methodology. **Wu Wei:** Validation. **Zeshan Abbas:** Writing – review & editing, Investigation, Formal analysis.

## Declaration of competing interest

We declare that we have no financial and personal relationships with other people or organizations that can inappropriately influence our work, there is no professional or other personal interest of any nature or kind in any product, service and/or company that could be construed as influencing the position presented in, or the review of, the manuscript entitled, “Operation parameters investigation on combustion and emission of a non-road diesel Engine based on orthogonal experiment design at different altitudes”.
